# Hypoxia Independently Induces AID Expression in CH12 B Cells

**DOI:** 10.1002/eji.70208

**Published:** 2026-05-19

**Authors:** Vincent Heyer, Sandy Haidar Ahmad, Mira Haddad, Inès Argentieri, Coraline Gessier, Bernardo Reina‐San‐Martin

**Affiliations:** ^1^ Institut De Génétique et De Biologie Moléculaire Et Cellulaire (IGBMC) Illkirch France; ^2^ Institut National de La Santé et De La Recherche Médicale (INSERM) Illkirch France; ^3^ Centre National de La Recherche Scientifique (CNRS) Illkirch France; ^4^ Université De Strasbourg Illkirch France

**Keywords:** AID, class switch recombination, hypoxia, hypoxia‐inducible transcription factor (HIF)

## Abstract

Hypoxia has been shown to shape the humoral immune response within germinal centers, where B cells diversify their receptors through somatic hypermutation (SHM) and class switch recombination (CSR). Both processes are initiated by activation‐induced cytidine deaminase (AID), which deaminates cytosines into uracils in immunoglobulin genes, leading to point mutations during SHM or to double‐stranded DNA breaks during CSR. Although AID's function is well characterized, the mechanisms governing its expression remain ill‐defined. We previously identified the hypoxia‐inducible transcription factor (HIF) transcription complex (comprised of HIF‐1α, HIF‐1β, and the auxiliary subunits HIF‐2α and HIF‐3α) as a regulator of optimal AID expression during CSR. Indeed, loss of HIF‐1α or HIF‐1β delays AID expression and impairs CSR. Here, we examine the contribution of HIF‐2α and HIF‐3α. We show that deficiency in HIF‐2α, but not HIF‐3α, impairs CSR, whereas combined loss of HIF‐1α and HIF‐2α does not exacerbate the defect, indicating that HIF‐1α is the dominant subunit driving this process. Importantly, we demonstrate that hypoxia can induce AID expression, independently of additional stimuli. Collectively, these findings reveal that hypoxia regulates AID expression in a context‐ and time‐dependent manner through HIF activation. This underscores the central role of hypoxic signaling in antibody diversification and suggests broader implications for immune regulation and the onset of B‐cell malignancies.

## Graphical abstract

Hypoxia activates the HIF complex (HIF‐1α, HIF‐1β, HIF‐2α) to induce AID expression in B cells independently of cytokine signaling. However, hypoxia alone is insufficient to trigger class switch recombination (CSR), which additionally requires cytokine‐driven chromatin accessibility, highlighting a mechanistic uncoupling between AID induction and functional antibody diversification.

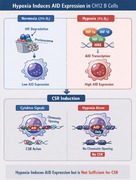

**Abbreviations**
AID: Activation‐induced cytidine deaminase
CSR: class switch recombination
HIF: hypoxia‐inducible transcription factor
HREs: hypoxia response elements
pVHL: von Hippel‐Lindau
SHM: somatic hypermutation

## 1 Introduction

During immune responses, in germinal centers, B cells diversify their receptors through somatic hypermutation (SHM) and class switch recombination (CSR). SHM introduces mutations in the variable regions of immunoglobulin genes to fine‐tune antibody specificity, while CSR changes the antibody isotype (from IgM to IgG, IgE, or IgA), altering effector functions [1]. Both processes are initiated by activation‐induced cytidine deaminase (AID), which converts cytosines to uracils in DNA. These lesions are processed by uracil DNA glycosylase (UNG) and mismatch repair proteins, resulting in mutations or double‐stranded DNA breaks [1]. Although crucial for producing high‐affinity antibodies, these mechanisms carry an inherent oncogenic risk, contributing to germinal center‐derived B‐cell lymphomas [2].

Cellular adaptation to low oxygen (hypoxia) is regulated by the hypoxia‐inducible transcription factor (HIF), a transcription factor composed primarily by the HIF‐1α and HIF‐1β and additional HIF‐2α and HIF‐3α minor subunits which can replace HIF‐1α [3]. Under normal oxygen conditions (normoxia), HIF‐1α is hydroxylated by prolyl hydroxylases (PHDs), recognized by von Hippel‐Lindau (pVHL) E3 ligase, and degraded by the proteasome [4]. During hypoxia, HIF‐1α stabilization occurs due to inhibited hydroxylation, allowing it to dimerize with HIF‐1β, translocate to the nucleus, and recruit coactivators such as p300/CBP [5]. The complex binds hypoxia response elements (HREs; RCGTG) in target gene promoters, activating transcription of genes that help cells survive and adapt to a low‐oxygen environment.

It has been shown that during immune responses, regions of hypoxia are observed within germinal centers and that the response to hypoxia modulates the humoral response [6, 7, 8]. We have previously implicated the HIF transcription factor complex in CSR [9]. Indeed, we have shown that genetic ablation of the genes encoding for the HIF‐1α and HIF‐1β subunits results in a CSR defect in CH12 cells, which is due to delayed AID expression [9]. The CSR defect observed can be rescued by overexpression of AID, demonstrating the role of the HIF complex in driving AID expression at the mRNA level and promoting efficient CSR [9]. Furthermore, the inactivation of the pVHL gene, which normally promotes HIF‐1α degradation, results in its accumulation and increases the expression of APOBEC3 family cytidine deaminases, including AID [10]. Likewise, B cells cultured under low oxygen (1% O_2_) display faster CSR than those in normal oxygen (21% O_2_), while high oxygen (60% O_2_) reduces IgG1 production [6]. Together, these findings indicate that oxygen tension modulates AID expression and isotype switching. Here, we explore the role of the additional subunits of the HIF complex (HIF‐2α and HIF‐3α) in driving AID expression and promoting CSR. Furthermore, we investigate whether hypoxia, *per se*, can trigger AID expression.

## 2 Results and Discussion

### 2.1 HIF‐2α but Not HIF‐3α Is Involved in Promoting AID Expression During CSR in CH12 B Cells

**FIGURE 1:**
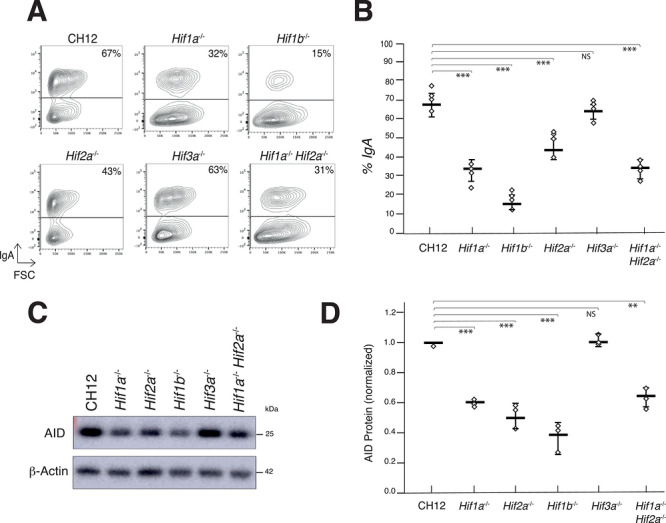
The HIF‐2α subunit contributes to AID expression and CSR. (A) Flow cytometry analysis of IgA expression in wild‐type (CH12), *Hif1a^−/−^, Hif1b^−/−^, Hif2a^−/−^, Hif3a^−/−^*, and *Hif1a^−/−^Hif2a^−/−^* CH12 B cells after 72 h in culture in the presence of ant‐iCD40, IL‐4, and TGF‐β (CIT). Representative contour plots are shown. The percentage of IgA^+^ cells is indicated. (B) Scatter plot showing the percentage of IgA^+^ cells from four independent experiments in wild‐type (CH12), *Hif1a^−/−^, Hif1b^−/−^, Hif2a^−/−^, Hif3a^−/−^*, and *Hif1a^−/−^Hif2a^−/−^* CH12 B cells. (C) Western blot for AID and β‐actin in wild‐type (CH12), *Hif1a^−/−^, Hif1b^−/−^, Hif2a^−/−^, Hif3a^−/−^*, and *Hif1a^−/−^Hif2a^−/−^* CH12 B cells after 72 h in culture with CIT. Molecular weights (kDa) are indicated. (D) Scatter plot showing AID protein levels relative to WT (CH12, set to one) and normalized to β‐actin from three independent experiments in wild‐type (CH12), *Hif1a^−/−^, Hif1b^−/−^, Hif2a^−/−^, Hif3a^−/−^*, and *Hif1a^−/−^Hif2a^−/−^* CH12 B cells. For (B) and (D), *p*‐value (two‐tailed Student's *t*‐test vs. CH12: ***p < 0.001, **p < 0.01, NS: not significant). Mean and dispersion are shown as horizontal and vertical lines, respectively. Uncropped images corresponding to (C) are presented in Figure S2.

We have previously shown that the HIF‐1α and HIF‐1β subunits play an essential role in regulating CSR by ensuring adequate and temporally controlled expression of AID [9]. However, our results indicate that CSR is not completely abolished in *Hif1a*‐deficient B cells, suggesting that the HIF‐2α and/or HIF‐3α subunits may be able to compensate for the absence of HIF‐1α. To date, the role of HIF‐2α in B cells remains poorly defined. Some studies suggest a potential involvement of this subunit in the transcriptional regulation of genes associated with the adaptive immune response, but the precise mechanisms remain unclear [7]. As for HIF‐3α, its role in B cells has not yet been characterized. To investigate the hypothesis of functional compensation and clarify the specific roles of the HIF‐2α and HIF‐3α subunits of the HIF transcription factor complex in promoting AID expression and regulating CSR, we inactivated the *Hif2a* and *Hif3a* genes using CRISPR/Cas9 genome editing in the murine CH12 cell line (Figure S1A,B), a well‐established model for studying CSR [11]. We find that inactivation of *Hif2a*, but not *Hif3a*, results in defective CSR (Figure 1A,B). Consistent with the CSR phenotype, AID protein levels were reduced in *Hif2a^−/−^* cells (Figure 1C,D), suggesting that HIF‐2α, like HIF‐1α, contributes directly or indirectly to the regulation of AID transcription and CSR. Nevertheless, combined inactivation of *Hif1a* and *Hif2a* did not result in a cumulative defect in CSR or AID expression (Figure 1A–D). Flow cytometry analysis confirmed that all generated knockout cell lines were IgM^+^ prior to stimulation (Figure S1C), ruling out the possibility of expansion of pre‐existing IgA^+^ cells. Re‐expression of HIF‐2α restored CSR efficiency to levels comparable to wild‐type cells (Figure S1D), confirming that the observed phenotype is specifically due to *Hif2a* deficiency. Furthermore, flow cytometry analysis of HIF‐deficient CH12 cells showed morphological changes (Figure 1A), possibly linking the role of the HIF complex to the previously described metabolic reprogramming of B cells [12]. Our results suggest that HIF‐1α plays a dominant role in regulating CSR. While HIF‐2α contributes to the process, it does not function independently of HIF‐1α. This observation raises the possibility that different HIF family members may have distinct but partially overlapping roles in regulating CSR. We conclude that HIF‐1α together with HIF‐1β are the main subunits of the HIF complex driving AID expression and operating during CSR and that the HIF‐3α subunit, unlike HIF‐1α and HIF‐2α, does not play a significant role in the transcriptional or functional control of CSR.

### 2.2 Hypoxia Potentiates and Is Sufficient to Induce AID Expression in CH12 B Cells

**FIGURE 2:**
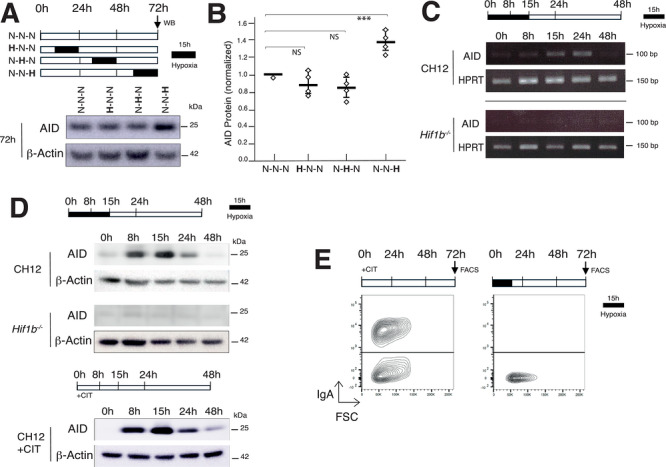
Exposure to hypoxia is sufficient to induce AID expression in CH12 B cells. (A) CH12 cells were cultured for 72 h in the presence of ant‐iCD40, IL‐4, and TGF‐β (CIT) under normoxia (N) or exposed to hypoxia (H; 3% O_2_ for 15 h) on Day 1 (H‐N‐N), Day 2 (N‐H‐N), or Day 3 (N‐N‐H). Western blot analysis for AID and β‐actin at 72 h is shown. Molecular weights (kDa) are indicated. (B) Scatter plot showing AID protein levels relative to WT (CH12, set to one) and normalized to β‐actin from four independent experiments. Mean and dispersion are shown as horizontal and vertical lines, respectively. *p*‐value (two‐tailed Student's *t*‐test: ***p < 0.001, NS: not significant). (C) RT‐PCR for AID and HPRT transcripts in WT (CH12) and *Hif1b^−/−^* CH12 B cells exposed to hypoxia (3% O_2_ for 15 h), then returned to normoxia for a total of 48 h. (D) Western blot analysis for AID and β‐actin expression in WT (CH12) and *Hif1b^−/−^* CH12 B cells exposed to hypoxia (3% O_2_ for 15 h), then returned to normoxia for a total of 48 h. For comparison, CIT‐stimulated CH12 cells (in the absence of hypoxia) are shown. (E) Flow cytometry analysis of CH12 cells cultured in the presence of ant‐iCD40, IL‐4, and TGF‐β (CIT) or just exposed to hypoxia for 15 h. Representative plots from three independent experiments are shown. The percentage of IgA^+^ cells is indicated. Uncropped images corresponding to (A), (C), and (D) are presented in Figure S2.

To determine whether hypoxia modulates AID protein expression, CH12 B cells were induced to undergo CSR in the presence or absence of hypoxia (15 h; 3% O_2_) at different time points on the first, second, or third day of culture (Figure 2A). In CH12 B cells, cytokine stimulation induces robust AID expression, reaching a maximal level around 16 h post‐stimulation and progressively decreasing after 48 h [9]. We find that exposing CH12 B cells to hypoxia on the third day of culture significantly enhances AID protein levels (Figure 2A,B). In contrast, hypoxia applied during the first or second day had no significant impact on AID protein levels (Figure 2A,B), most likely due to the robust cytokine‐driven AID expression at these stages. As hypoxia rapidly triggers nuclear accumulation of the HIF complex within a few hours [13], this experimental design allowed assessment of its effect on AID protein levels after 72 h, corresponding to the phase when cytokine‐induced AID expression normally declines. We conclude that exposing B cells to hypoxia at Day 3 boosts the level of AID expression and suggests that hypoxia, *per se*, might be sufficient to trigger AID expression.

To assess whether hypoxia alone can trigger transcription of the *Aicda* gene, we subjected CH12 B cells to hypoxia (3% O_2_) for 15 h in the absence of cytokine stimulation, after which cells were returned to normoxia for a total incubation period of 48 h (Figure 2C,D). We then assessed by RT‐PCR (Figure 2C) and western blot (Figure 2D) whether under these conditions, AID expression could be induced. We find that exposing CH12 B cells to hypoxia (in the absence of other stimuli) triggers the expression of AID both at the mRNA and protein levels (Figure 2C,D). Importantly, AID induction was dependent on HIF‐1β, the main subunit of the HIF complex (Figure 2C,D). A similar result would be expected from inactivation of *Hif1a* and/or *Hif2a*. For comparison, cytokine‐stimulated wild‐type CH12 cells were included as a reference (Figure 2D). Despite being able to trigger AID expression, hypoxia by itself was unable to trigger CSR (Figure 2E). We conclude that hypoxia, *per se*, in the absence of additional stimuli, can trigger AID expression. Nevertheless, it cannot independently initiate the full transcriptional program required to open the IgH locus for cytidine deamination by AID and to trigger the subsequent enzymatic cascade that is necessary to successfully undergo CSR.

Isotype switching is a central process in the adaptive immune response, dependent on the precise and temporally regulated expression of the enzyme AID. Understanding the factors that control AID is therefore essential to elucidate the mechanisms that enable B cells to diversify their antibodies. HIF transcription factors, known for their role in the response to hypoxia, are emerging as potential regulators of this process, but their specific contribution in B cells has remained poorly defined.

Our findings provide mechanistic insights into the role of the HIF transcription factor in CSR regulation. Targeted disruption of *Hif1a* or *Hif2a* markedly attenuated both AID expression and CSR, whereas *Hif3a* inactivation had no significant effect. These results indicate that HIF‐1α and HIF‐2α function in a complementary manner to sustain adequate AID levels, suggesting the existence of a functional hierarchy within the HIF family in controlling CSR, the main subunits being HIF‐1α and HIF‐1β.

Although hypoxia signaling has recently been linked to germinal center metabolism and antibody responses, the molecular mechanisms connecting oxygen sensing to the core machinery that initiates antibody diversification remain poorly defined. Recent work has shown that HIF‐1α regulates CSR to IgA in vivo through metabolic reprogramming of B cells [12], highlighting an important link between oxygen sensing, cellular metabolism, and humoral immunity. Our findings complement this study by identifying AID expression itself as a target of HIF‐dependent regulation. While HIF‐1α influences metabolic programs that affect CSR in vivo [12], our results indicate that the HIF transcriptional complex also modulates CSR by contributing to the transcriptional induction of AID.

Together, these findings suggest that HIF signaling may control antibody diversification through two interconnected mechanisms: by reshaping the metabolic state of activated B cells and by regulating the expression of AID. In this framework, metabolic changes induced by HIF activity could indirectly influence AID expression, while direct transcriptional regulation of the *Aicda* locus may also contribute. Future studies will therefore be required to determine the relative importance of direct transcriptional regulation (e.g., binding of the HIF complex to the *Aicda* promoter in B cells), as reported for other cell types [14] versus metabolic mechanisms in controlling AID expression.

An additional important question concerns the role of these mechanisms under physiological hypoxic conditions. Given that HIF‐1β is required for hypoxia‐induced AID expression, our results predict that inactivation of HIF‐1α and/or HIF‐2α would impair the induction of AID under hypoxic conditions in vivo. Elucidating how hypoxia‐dependent signaling integrates with the molecular control of AID expression will therefore be important for understanding how microenvironmental cues influence the initiation of antibody diversification. Together, these observations identify AID expression as a previously unrecognized node through which hypoxia signaling can influence the initiation of antibody diversification.

We have shown that exposing B cells to hypoxia alone is sufficient to induce AID expression but fails to elicit effective CSR. When hypoxia is applied to cytokine‐stimulated cells, however, it enhances and extends AID expression, highlighting a modulatory and context‐dependent role of the hypoxic microenvironment in CSR regulation. This has implications for the dynamics of AID expression in vivo within germinal centers.

These observations also have important implications for tumorigenesis. Tumor hypoxia, a hallmark of many solid malignancies, leads to chronic HIF activation. We have shown that hypoxia *per se* can trigger AID expression in CH12 B cells. Interestingly, it has been reported that in fibroblast spheroid cultures exposed to hypoxia, HIF‐1α activation coincides with AID induction and the expression of reprogramming genes such as *Oct4* and *Dnmt1* [14]. Understanding the relationship between HIF and AID in hypoxic conditions may therefore provide insights into both immune regulation and cancer development. If hypoxia‐mediated induction of AID occurs in this setting, it could promote genomic instability and mutagenesis in tumor cells, thereby facilitating tumor progression. Elucidating the molecular interplay between hypoxia, HIF signaling, and aberrant AID expression may thus advance our understanding of the onset of cancer and may reveal novel therapeutic targets.

## 3 Materials and Methods

### 3.1 Cell Culture

CH12 cells were cultured in RPMI 1640 (Gibco) with 10 mM HEPES, 10% heat‐inactivated fetal calf serum (Bodinco BV), 1 mM sodium pyruvate, 100 U/mL penicillin/streptomycin, and 50 µM β‐mercaptoethanol. Hypoxia was induced by culturing CH12 cells in 3% O_2_ for 15 h.

### 3.2 CRISPR/Cas9

CH12 cells were transfected with a plasmid expressing high‐fidelity Cas9 (Cas9‐HF) fused to EGFP, co‐expressing two guide RNAs flanking exon 2 of the *Hif2a* or *Hif3a* genes (Figure S1A,B)—gRNAs: *Hif2a* (GGAGAATTGACTCACTGTGG and ATTTATTTAGTTGCGTGTGA) and *Hif3a* (CACGCGACAGGTCGCACGT and GTATCCCATGGCTAGAAAGG). Twenty‐four hours after transfection, single CH12 cells expressing Cas9‐HF (EGFP^+^) were sorted by FACS (Aria Fusion, BD) into 96‐well plates and cultured for 2 weeks. The clones obtained were genotyped by PCR using specific primers (*Hif2a*‐F: TCTGACCGTAGCTTCTTCGC, *Hif2a*‐R: GCCAGAGGTCCAGAGGTACT, *Hif3a*‐F: GGCTCCAAGGGGAGAAAACTG, *Hif3a*‐R: GACTGGTCCAGCACACGAAG). Deletion of exon 2 was confirmed by sanger sequencing.

### 3.3 Western Blot

Total cell extracts were prepared using standard methods (RIPA). Proteins were separated by SDS‐PAGE and transferred to PVDF membranes (Millipore). Antibodies used were anti‐AID (1/10,000, IGBMC) and anti‐β‐actin (1/20,000, Sigma‐Aldrich).

### 3.4 CSR Assays

CH12 cells were cultured for 72 h in vitro in the presence of IL‐4 (5 µg/µL, Peprotech), TGF‐β (1 µg/µL, R&D Systems Europe), and an anti‐CD40 antibody (200 ng/mL, eBioscience). IgA and IgM expression was assessed by labeling cells with an anti‐IgA‐PE antibody (1/500, Southern Biotech) or an anti‐IgM‐FITC antibody (1/500, Jackson ImmunoResearch). Prior to analysis, cells were incubated with DAPI (0.1 µg/mL) to discriminate dead cells. Samples were then analyzed by flow cytometry (FACS BD SYMPHONY A1) and using the FlowJo software.

### 3.5 Retroviral Transductions

Retroviral supernatants were generated by transfecting Bosc23 cells with a construct encoding HIF‐2α and used to transduce *Hif2a* ^−/−^ CH12 cells. Transduced cells were selected with puromycin (1 µg/mL) and assayed for CSR by flow cytometry.

### 3.6 RT‐PCR

RNA extraction was performed according to the Nucleospin RNA protocol (MN 740955, Macherey‐Nagel), and cDNA synthesis was performed using standard methods (Superscript IV, Thermo). RT‐PCR was performed with Taq Phusion (Thermo, F‐565) over 25 cycles and the following primers: AID‐F (GAAAGTCACGCTGGAGACCG), AID‐R (TCTCATGCCGTCCCTTGG), HPRT‐F (GTTGGATACAGGCCAGACTTTGTTG), and HPRT‐R (GATTCAACTTGCGCTCATCTTAGGC).

## Author Contributions

Conceptualization and methodology: Vincent Heyer and Bernardo Reina‐San‐Martin. Investigation: Vincent Heyer, Sandy Haidar Ahmad, Mira Haddad, Inès Argentieri, and Coraline Gessier. Writing – original draft: Vincent Heyer, Sandy Haidar Ahmad, and Bernardo Reina‐San‐Martin. Writing, review, and editing: Vincent Heyer, Sandy Haidar Ahmad, and Bernardo Reina‐San‐Martin. Supervision and Funding acquisition: Bernardo Reina‐San‐Martin.

## Conflicts of Interest

The authors declare no conflicts of interest.

## Supporting information




**Supporting File**: eji70208‐sup‐0001‐Figures.pdf.

## Data Availability

The data that support the findings of this study are available from the corresponding author upon request.
