# Supplementary material for: Hypoxia Independently Induces AID Expression in CH12 B Cells
Source: Eur J Immunol. 2026 May 19;56:e70208. doi: 10.1002/eji.70208 (PMC13185679; doi:10.1002/eji.70208)
Supplement: Supplementary file 1 — Supporting File: eji70208‐sup‐0001‐Figures.pdf. [file EJI-56-e70208-s001.pdf]

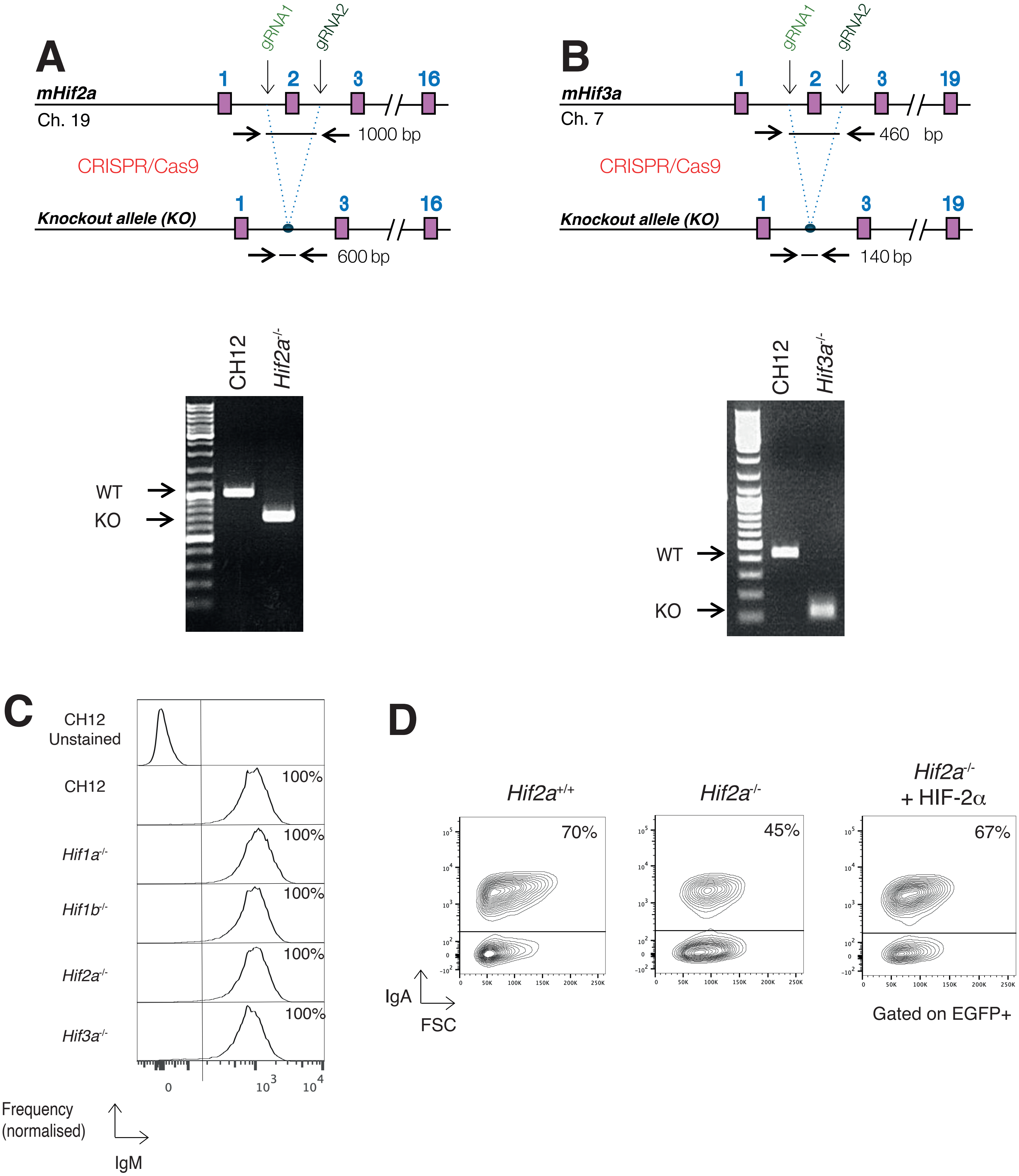

Figure S1. CRISPR/Cas9 knockout and genotyping strategy by PCR for (**A**) *Hif2a* and (**B**) *Hif3a*. gRNAs flanking a critical exon and expected PCR products are indicated. (**C**) Flow cytometry analysis of IgM expression in unstimulated Wildtype (CH12, unstained and stained), *Hif1a*$^{-/-}$, *Hif1b*$^{-/-}$, *Hif2a*$^{-/-}$ and *Hif3a*$^{-/-}$ cells. The Y axis represents the frequency of normalized cell counts across samples. (**D**) Flow cytometry analysis of IgA expression in Wildtype (CH12) and *Hif2a*$^{-/-}$ CH12 cells reconstituted or not with a retrovirus expressing HIF-2α after 72h in culture in the presence of CD40L, IL-4 and TGFβ (CIT). Transduced cells were gated on EGFP expression. Representative contour plots from three independent experiments are shown. The percentage of IgA+ cells is indicated.

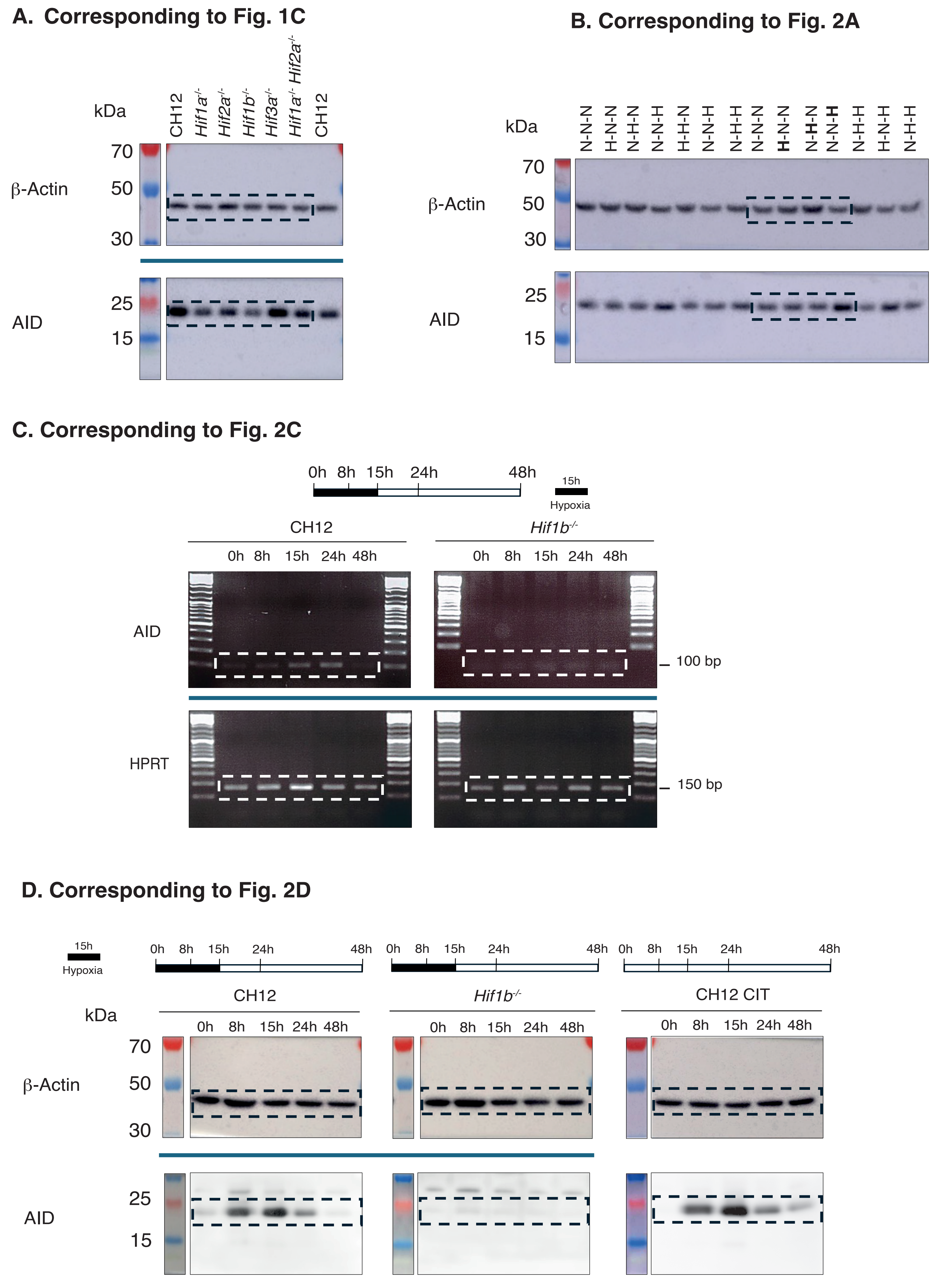

Figure S2. Uncropped Western blots and agarose gels. Molecular weights (kDa or bp) are indicated. For Western blot, membranes were cut and incubated independently with indicated antibodies. Sections shown in corresponding figures are indicated by a dashed box.
